# Natural Compounds as Medical Strategies in the Prevention and Treatment of Psychiatric Disorders Seen in Neurological Diseases

**DOI:** 10.3389/fphar.2021.669638

**Published:** 2021-05-13

**Authors:** Esra Küpeli Akkol, Irem Tatlı Çankaya, Gökçe Şeker Karatoprak, Elif Carpar, Eduardo Sobarzo-Sánchez, Raffaele Capasso

**Affiliations:** ^1^ Department of Pharmacognosy, Faculty of Pharmacy, Gazi University, Ankara, Turkey; ^2^ Department of Pharmaceutical Botany, Faculty of Pharmacy, Hacettepe University, Ankara, Turkey; ^3^ Department of Pharmacognosy, Faculty of Pharmacy, Erciyes University, Kayseri, Turkey; ^4^ Department of Psychiatry, Private French La Paix Hospital, Istanbul, Turkey; ^5^ Instituto de Investigación y Postgrado, Facultad de Ciencias de la Salud, Universidad Central de Chile, Santiago, Chile; ^6^ Department of Organic Chemistry, Faculty of Pharmacy, University of Santiago de Compostela, Santiago de Compostela, Spain; ^7^ Department of Agricultural Sciences, University of Naples Federico II, Potici, Italy

**Keywords:** Alzheimer's diseases, anxiety, depression, Parkinson's disease, natural compound

## Abstract

Psychiatric disorders are frequently encountered in many neurological disorders, such as Alzheimer’s and Parkinson diseases along with epilepsy, migraine, essential tremors, and stroke. The most common comorbid diagnoses in neurological diseases are depression and anxiety disorders along with cognitive impairment. Whether the underlying reason is due to common neurochemical mechanisms or loss of previous functioning level, comorbidities are often overlooked. Various treatment options are available, such as pharmacological treatments, cognitive-behavioral therapy, somatic interventions, or electroconvulsive therapy. However oral antidepressant therapy may have some disadvantages, such as interaction with other medications, low tolerability due to side effects, and low efficiency. Natural compounds of plant origin are extensively researched to find a better and safer alternative treatment. Experimental studies have shown that phytochemicals such as alkaloids, terpenes, flavonoids, phenolic acids as well as lipids have significant potential in *in vitro* and *in vivo* models of psychiatric disorders. In this review, various efficacy of natural products in *in vitro* and *in vivo* studies on neuroprotective and their roles in psychiatric disorders are examined and their neuro-therapeutic potentials are shed light.

## Introduction

Psychiatric symptoms seen in neurological diseases have brought with them a multitude of conceptual difficulties. The increasing rate in the detection of mental health problems is usually attributed to a broadening of comprehension and advanced treatment techniques of neurological disorders as well as lengthened life expectancy, hence understanding the differences and similarities between these disorders is now relevant to investigations beyond a single discipline (WHO Fact Sheet, 2001). Clinical presentations of various psychiatric manifestations may be suspected according to the underlying neurological diseases. Moreover, an inattentive examination may easily lead to misdiagnosis or mistreatment. In the case of comorbidity, good knowledge about the overlapping disease mechanism is necessary for appropriate management (Lyketsos et al., 2007). These psychiatric comorbidities increase the disease burden and make the management of combined diseases difficult. The development of psychiatric symptoms has been associated with altered neurotransmitter signaling, changes in the brain structure, unstable inflammatory substance levels, and impaired neurotrophic factors.

Natural products have a wide range of pharmacological or biological activity making them proper candidates for the treatment of neurological disorders, neurodegenerative diseases, and related psychiatric disorders (Harvey et al., 2010). Many studies on the identification and discovery of new neuroprotective drugs have displayed that natural bioactive compounds have possible as neuroprotective agents against various types of neurodegenerative disorders. They exhibit their neuroprotective effects through different mechanisms. It is known that secondary metabolites belonging to many classes of chemical compounds, especially diterpenes and cyclodepsipeptides, interact with the γ-aminobutyric acid (GABA)-A receptor. Secondary metabolites that positively affect the binding of muscimol to the GABA receptor complex are alkaloids. Similarly, the tendency of some flavonoids to bind to the benzodiazepine site on the GABAA receptor has been demonstrated, and their anti-inflammatory and antioxidant effects also contribute to the activity (Rehman et al., 2019). They have also been demonstrated to modulate multiple signaling pathways through direct effects on enzymes such as kinases, regulatory receptors, and proteins (Bagli et al., 2016; Wang et al., 2017).

In this paper, we aimed to discuss and further propose novel natural treatment modalities that can be utilized in the prevention and treatment of psychiatric disorders, most commonly anxiety and depressive disorders in the background of common neurological diseases namely Alzheimer’s disease and Parkinson’s disease.

## Epidemiology and Etiology of Neurological Disorders

In so far as lifetime occurrence is to be considered, neurological diseases represent a significant load of chronic disability and financial burden globally especially in the older age group (Whiteford et al., 2015). As the aging population increases consistently, the prevalence of most neurological disorders has increased dramatically from the years 1990–2016 with a rate up to 117% (Nichols et al., 2019). Two of the most commonly seen neurological disorders are Alzheimer’s disease (AD) and Parkinson’s disease (PD) which are neurodegenerative in nature. AD affects about 6–10% of people over 65 years (Burns and Iliffe, 2009; Hebert et al., 2013). This disease is associated with neuroinflammation and atrophy with extracellular amyloid plaques and intraneuronal tau protein neurofibrillary tangles in brain tissue. The exact etiology of the neurodegenerative disorder remains unclear, but it is most likely to be resulting from both genetic and environmental factors interactively (Jiang et al., 2013). Clinically, commonly encountered symptoms are a significant intellectual decline from a previously higher cognitive level, memory loss, disorientation to time and place, behavioral disturbances, personality changes, and loss of interest in previously joyful activities. The onset of symptoms is frequently gradual and they usually interfere with patients' ability to perform everyday activities (Alzheimer's Disease, 2016).

Parkinson’s disease is reported to affect approximately 1% of the population above 60 years (Ascherio and Schwarzschild, 2016). The onset of the disease concurs with 65–70 years old, but genetic variants can be seen earlier across the life span. In population-based cohorts, the symptoms are reported to start before the age of 40 in less than 5% of cases (Tysnes and Storstein, 2017). Similar to AD, in PD determining a single causative agent is not possible because of the multifactorial etiology. Degeneration in the brain occurs with loss of dopaminergic cells in the substantia nigra in the midbrain, and accumulation of Lewy bodies in the neurons histopathologically (Baltazar et al., 2014). The clinical presentation typically involves abnormal posture, flat affected mask face, and extensive movement disturbances with muscle rigidity (Pagonabarraga et al., 2015).

## Co-Morbidity of Psychiatric Disorders in Neurological Diseases

Psychiatric disorders are frequently encountered in many neurological disorders, for instance, AD and PD along with epilepsy, migraine, essential tremors, and stroke (Penner and Paul, 2017). These psychiatric comorbidities increase the burden of disease and render the management of combined disorders difficult. Altered signaling of neurotransmitters, changes in brain structure, imbalanced levels of inflammatory substances, disrupted neurotrophic factors, psychosocial agents, and pain have been accused in the development of psychiatric symptoms accompanying neurological diseases (Hesdorffer, 2016). Whether the underlying reason is due to common neurochemical mechanisms or loss of previous functioning level, comorbidities are often overlooked. Because these complicated cases have salient psychological and neurocognitive components, their assessment requires the combined efforts of neurologists to assess musculoskeletal features, as well as psychiatrists for psychological evaluations. A detailed approach by experienced neuropsychiatry teams is necessary, which is not always achievable. The most common comorbid diagnoses in neurological diseases are depression and anxiety disorders along with cognitive impairment (Mini and Alcohol, 2007). Psychotic symptoms also prevail among severe clinical pictures, however, this topic will not be included in this paper (Chang and Fox, 2016).

Similar to other types of dementia, depression is one of the most common psychiatric disorders accompanying AD. Depending on the diagnostic method, the severity of disease, and sampling methods in various study populations, up to 50% of patients with AD were reported to demonstrate depression at least once throughout the disease course (Ryu et al., 2017). Studies revealed that comorbid depression is related to more disability in daily living activities, hence higher nursing home placement rate, worse quality of life, faster cognitive decline with higher mortality, and higher depression rate among caregivers in patients with AD (Starkstein et al., 2008). It is not always straightforward to differentiate depression from one of the cardinal symptoms of AD, apathy. In fact, these presentations may overlap and complicate the diagnostic process in the majority of the cases (Kim et al., 2019). Anxiety is another psychiatric appearance commonly associated with AD and the pooled prevalence rate can be as high as 39% (Zhao et al., 2016). Anxiety disorders are related to other psychiatric morbidities, disability, increased healthcare admissions, and mortality in patients with AD, as in the general adult population. However recent studies are focused beyond the cross-sectional association and even established ground for deterministic causality interactions. Recent findings provide innovative advancements in the literature by demonstrating data of anxiety in the younger age as a risk factor for AD in later life (Becker et al., 2018). This is supported by a recent metanalysis in which the authors recommend that anxiety is marginally associated with an increased risk of AD (Santabárbara et al., 2020).

Psychiatric disorders are commonly perceived in Parkinson's disease (PD), with anxiety and mood disorders. Depression occurs in as high as 35% of patients with PD (Aarsland et al., 2012). Similarly, anxiety disorders, principally comprehensive social phobia, panic, and anxiety, occur in up to 40% of the patients (Katunina and Titova, 2017). Evidence beyond psychological factors suggests that both anxiety and depression may have pathophysiological links with PD because these mood disorders are more common in individuals with PD than in patients with other chronic conditions (Martínez-Martín and Damián, 2010).

## Common Mechanisms of Pathophysiology in Neurological and Psychiatric Disorders

**FIGURE 1:**
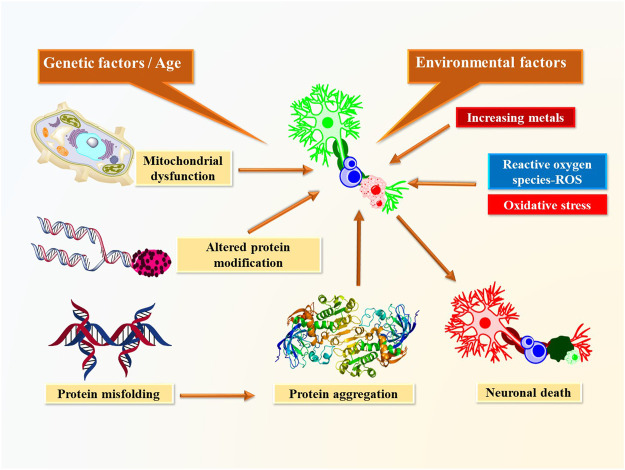
Mechanism involve in development of neurodegenerative disorders.

Neurodegenerative disorders such as Alzheimer's disease, Parkinson's disease, and Huntington's disease (HD) make up a group of pathologies described by various etiologies with distinct pathophysiological and morphological features. These disorders arise through multifactorial conditions such as oxidative stress and free radical formation, protein misfolding with defective protein aggregation and degradation, exposure to pesticides and metal toxicity, and damaged bioenergetics and mitochondrial dysfunction (Figure 1).

### Anxiety Disorders

**FIGURE 2:**
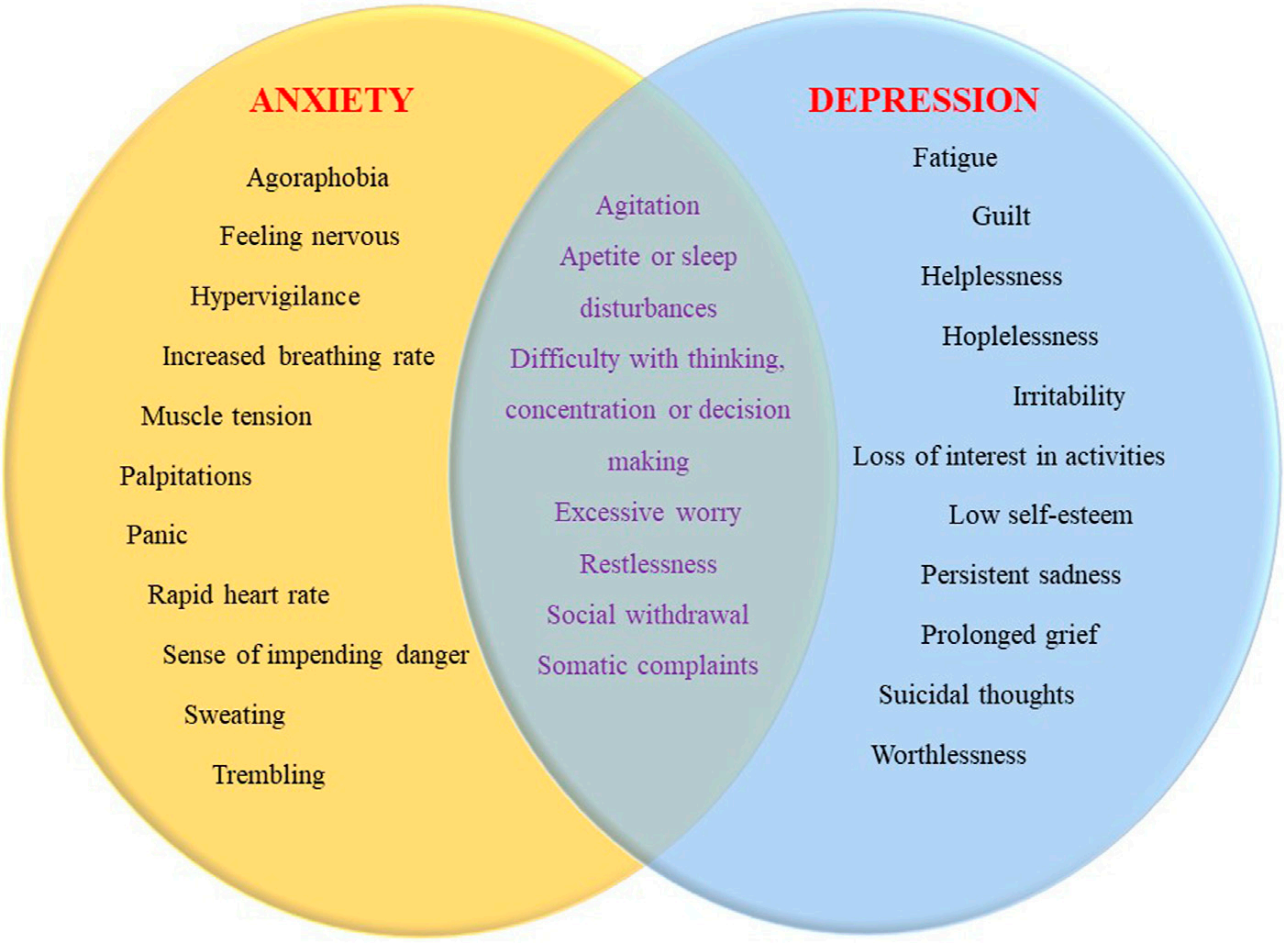
Symptom overlap between anxiety and depression.

The definition of anxiety is a feeling often accompanied by subjective unpleasant emotions, along with somatic complaints, rumination, and restless movements, often characterized by fear and worry about the future (Figure 2) (Newman et al., 2013). Anxiety disorders, on the other hand, refer to pathological conditions of patients who meet the appropriate standards for a specific period of time based on certain diagnostic criteria. In this case, the severity of anxiety is excessive, it occurs in an inappropriate context and long in duration (Andrews et al., 2010). The incidence of anxiety disorders is approximately 10–14% and the lifetime prevalence is almost as high as 30%. Female predominance stands out with a two-fold increase in the rate, and disease onset is usually before the age of 30 (Bandelow and Michaelis, 2015). The most common subtype is a specific phobia and the second is a social anxiety disorder. As with most psychiatric disorders, anxiety disorders are affected by a complex combination of environmental and genetic factors. To receive the diagnosis, the symptoms must be severe and disrupt daily life activities. Medical conditions that can cause anxiety include heart disease, hyperthyroidism, alcohol, caffeine, or cannabis use, and withdrawal from certain medications (McLean et al., 2011). Dysfunctions and overstimulation in the neural circuit involving the amygdala and hippocampus which maintains HPA axis control over the sympathetic nervous system regulates emotions and establishes emotional memory are thought to play the main role in the formation of anxiety (Dresler et al., 2013). The influence of neurotransmitters like serotonin and norepinephrine has been implicated and constructed as the basis for treatment strategies (Nuss, 2015). Novel studies have broadened the perspective into specific cell groups in amygdala nuclei as well as drew attention to CRH interactions with the help of neuroimaging studies (Hamati, 2018). The fundamental concept underlying the psychological theories is that anxiety is an overwhelmed reaction of the human psyche against situations of uncertainty (Bateson et al., 2011).

In the last decades, anxiety has been shown to significantly correlate with cognitive impairment from all severity levels (Regan and Varanelli, 2013). It is not surprising that memory problems may directly result in feelings of uncertainty (hence anxiety) in an affected individual. Another finding that supports this notion comes from studies evaluating the treatment effect of antidementia medications which are shown to alleviate anxiety symptoms while treating AD (Cacabelos, 2007). Anxiety has also been suggested to be a catastrophic reaction against the disclosure of the diagnosis of AD to the patient (Pinner and Bouman, 2003). Some clinical issues are recommended to be considered when distinguishing anxiety disorders accompanying AD from other comorbid conditions. A vast majority of anxiety symptoms are experienced in the cognitive domain such as catastrophic thinking and impending doom. Therefore they cannot be easily assessed using standard self-report measures due to cognitive destruction in the context of AD (Poulin et al., 2011). Moreover, many anxiety symptoms are non-specific and overlap with symptoms of other psychiatric syndromes that are common in dementia, especially depression and agitation (Leyhe et al., 2017). Also, other psychiatric conditions with anxiety symptoms are reasonably common in the older population without AD, which makes the diagnostic process more challenging (Balsamo et al., 2018).

The most frequently seen anxiety disorder subtypes in PD are panic disorder and generalized anxiety disorder (Hanna and Cronin-Golomb, 2012). Similar to other chronic conditions, patients with PD experience significantly higher stress than people without any diseases. This stress can be explained as the fear of stigmatization by the public due to overt movement disturbances or may be related to the neurochemical environment of the brain itself (Kudlicka et al., 2011). The imbalance of neurotransmitters like serotonin, norepinephrine, GABA, and dopamine has been implicated to play a role in the pathogenesis of anxiety. A region in the hindbrain called locus cereleus controls norepinephrine function and adjusts alertness, therefore has been thought to play a role in anxiety and fear (McCall et al., 2017). Dopaminergic neuron death in PD is thought to be directly responsible for anxiety formation. Physiologically dopamine inhibits the activation of neurons from locus cereleus, and loss of dopaminergic inhibition can result in anxiety (Leentjens, 2012). In addition, antiparkinsonian drugs may take part in the pathogenesis of anxiety. Anxiety symptoms seen after dose adjustment or medication switch in the treatment process may be an important expression of levodopa fluctuations (Seppi et al., 2019). Commencing on dopaminergic drugs to treat symptoms of PD is associated with addictive behaviors and impulse control disorders, and may cause the underlying anxiety to be underdiagnosed (Ambermoon et al., 2011).

### Depressive Disorders

Depression is a leading cause of disability globally and is a major contributor to the overall global burden of disease as well as significant economical burden worldwide (Lépine and Briley, 2011). The typical clinical picture includes psychological, physical, and behavioral symptoms such as feelings of worthlessness, loss of interest in daily activities, depressed mood, guilt, fatigue, difficulty in concentration, suicidal thoughts, alteration in sleep, weight, and appetite in most of the patients (Figure 2). Approximately 8–12% of the world's population experiences depression at least once in a lifetime (Fried et al., 2016). In addition to proposed candidate genes, according to the monoamine hypothesis, the lack of neurotransmitters may contribute to the occurrence of depression. The decrease in serotonin levels is linked to mood, sleep, and behavior regulation, while norepinephrine to fight or flight response and finally dopamine has been linked to movement, pleasure, and motivation (Shyn and Hamilton, 2010). Other etiological hypotheses put forward by novel technology appliances of neuroimaging methods and laboratory evaluations generally focus on the brain network, altered neuroplasticity, inflammation, and oxidative stress (Duman and Voleti, 2012). Diathesis-stress models assert that stressful experiences trigger depression in those who may be vulnerable because of the underlying biological and psychosocial characteristics. The trigger may involve an exaggerated grief response to a loss. For example, frequent depression can occur in older people due to the death of a loved one, a role change in retirement, or coping with a serious illness (Saveanu and Nemeroff, 2012).

Depressive disorder is one of the most common psychiatric comorbidities of both AD and PD. Some appearances cover full syndromal major depressive disorder, however, most patients have less severe forms of depression called dysthymia or subsyndromal depression (Rickards, 2006). The impact of the disease itself often serves as a significant stressor and may indicate loss of function in the population at risk. Due to the neurological disorder, social interactions are mostly interrupted, isolation may occur, which then renders the individual prone to depression (Rickards, 2005). Sometimes the neurological illness is thought to reflect a progressive impairment in cognitive abilities that support both the ability to communicate suicidal thoughts and depressive symptoms, making clinical evaluation difficult (Oguru et al., 2010). Alternatively, depression can occur in the prodromal period while symptoms specific to AD and PD have not yet appeared (Kales et al., 2015). These attacks are not adequately understood in their temporal features and may have distinct and atypical phenotypes different from idiopathic depression. Recent discussions to specific diagnostic criteria recommendations to identify depression, particularly as a separate entity in AD have risen gradually (Engedal et al., 2011). There is evidence that AD and PD patients with depression are largely helped by psychotherapy. If depression is not detected or treated with neurological disorders, this can cause persistence of symptoms and further disability. However, since anxiety disorders are frequently associated with depression, it is significant to manage both conditions at the same time (Enache et al., 2011). In addition to patients, caregivers are also at risk; a recent study reported that caregivers of AD and PD patients are more likely to experience depression than those who do not (Cupidi et al., 2012).

## Medical Treatment Strategies for Psychiatric Disorders Seen in Neurological Disorders

Anxiety and depressive disorders are well-studied psychiatric conditions with regard to management methods. Treatment strategies on the background of neurological diseases are similar to non-comorbid cases in terms of basic principles, except that extra caution needs to be considered in medication interactions (Leahy et al., 2011). Various treatment options are available, such as pharmacological treatments, cognitive-behavioral therapy, somatic interventions, or electroconvulsive therapy (Gorman, 1996). Some of the listed methods aim to improve anxiety and depression simultaneously, but some are specific to the individual disorder. Drugs such as benzodiazepines with anxiolytic properties are usually commenced for a short period to relieve anxiety, however, they are preferred as the last option because of their negative effect on cognitive conditions in AD and balance problems in PD (el-Guebaly et al., 2010).

Antidepressants are introduced as a treatment modality in depression and anxiety since the 1950s and are widely used with high success rates (Noel, 2015). Oral antidepressant therapy may have some disadvantages, such as interaction with other medications, low tolerability due to side effects, and low efficiency (Santarsieri and Schwartz, 2015). Except for special conditions, the first-generation antidepressants which act by increasing the extracellular usability of monoamines, namely tricyclic antidepressants and MAO inhibitors have been shelved in the last decades (Hillhouse and Porter, 2015). Although not without imperfection, new generation antidepressants are widely preferred in the treatment of depression and anxiety. Some of the side effects include sexual dysfunction, gastrointestinal effects, and discontinuation syndrome (Ramachandraih et al., 2011). These drugs act as selective serotonin reuptake inhibitors (SSRI) by increasing the amount of serotonin in the synaptic cleft. SSRI drugs are also effective in treating anxiety with varying doses. The side effect profile is generally worse when used for anxiety disorders and neurological diseases (Rickels et al., 2012). Although both diseases have abundant biological treatment options, psychotherapeutic interventions are used effectively in both (Weitz et al., 2018).

## Natural Compounds and the Affected Pathways in Similar Ways

### Alkaloids

Alkaloids are naturally consisting components from carbon, hydrogen, oxygen, and nitrogen molecules, and are found especially in flowering plants. Families like Amaryllidaceae, Papaveraceae, Solanaceae, and Ranunculaceae are largely rich in various types of alkaloids (Girdhar et al., 2015; Bayazeid et al., 2020).

Among the alkaloid compounds used in neurodegenerative diseases, there are indole, oxindole, pyrroloindole, isoquinoline, aporphine, pyridine, piperidine, *β*-carboline, vinca, and methylxanthene derivatives. These compounds are muscarinic and adenosine receptors agonists, anti-amyloid and MAO inhibitors, acetylcholinesterase (AChE) and butyrylcholinesterase (BChE) inhibitors, an inhibitor of α-synuclein aggregation, dopaminergic and nicotine agonists, and an N-methyl-d-aspartate antagonist (NMDA). Therefore they play positive roles in improving the pathophysiology of this kind of diseases, including Alzheimer's disease (AD), Parkinson's disease (PD), Huntington disease (HD), schizophrenia (S), epilepsy (E), and stroke (Table 1) (Ng et al., 2015; Hussain et al., 2018).

**TABLE 1:** Alkaloids, their sources, target neurodegenerative diseases and their mechanism of effects.

Alkaloids (Class of Alkaloids)	Plant-derived	Diseases	Mechanism of effect	References
Arecoline (Pyridine Alk.)	*Areca catechu* L.	AD	It acts as a muscarinic receptor agonist	Girdhar et al. (2015)
		S	It has been stated as a partial agonist of the acetylcholine muscarinic receptor. It has activity against schizophrenia by directly targeting OLs and also inhibits demyelination of white matter. It increases social and cognitive properties and maintains myelin damage in the cortex by facilitating differentiation of oligodendrocyte precursor cells by dephosphorylating the active protein kinase AMPK*α*. It demonstrates to enhance body excitability through stimulation of muscarinic receptors as well as enhancing memory and learning abilities	Hussain et al. (2018), Wang et al. (2015)
Acetylcorynoline (Isoquinoline Alk.)	*Corydalis bungeana* Turcz.	PD	It has neuroprotective activity by inhibiting dopaminergic neuron loss, aggregation of *α*-syn protein, and dopamine level depletion. It reduced apoptosis by decreasing the expression level of egg-laying abnormal-1, an apoptosis modulator. Protein aggregation and misfolding causing the production of inclusion bodies make an important contribution to the pathogenesis of PD. It may prevent pathogenesis by enhancing proteolysis by a somatic proteasome activity intervened by increasing the expression level of rpn-5, a proteasome regulatory subunit	Shahpiri et al. (2016)
Berberine (Isoquinoline Alk.)	*Hydrastis canadensis* L. *Coptis chinensis* Franch *Berberis aquifolium* Pursh *B. vulgaris* L. *B. aristata* DC	AD	By preventing hippocampal neurodegeneration, it developed behavioral disorder and decreased BACE-1 activity. It has monoamine oxidase and AChE inhibitory properties. In addition, it has been shown to attenuate the accumulation of A*β* plaques and inhibit the expression of BACE-1. It reduces the hyperphosphorylation of tau on calyculin-A treatment by restoring protein phosphatase-2A activity and inhibiting GSK-3*β* activation in HEK293 cells	Hussain et al. (2018), Mathew et al. (2014), Pohanka (2014), Yan and Vassar 2014)
		PD	By preventing neuronal damage of dopaminergic neurons, it increases motor stability and synchronization, thus, importantly prevents balance loss. It also develops short-term memory by preventing apoptosis and improving neurogenesis in the hippocampal dentate gyrus	Hussain et al. (2018), Huang et al. (2017)
		HD	It regulates autophagic function that improves movement coordination and motor function, which may be useful in clearing misfolded proteins in the HD state. With the activation of an autophagic function, also decreases mutant htt deposits and aggregation	Hussain et al. (2018), Jiang et al. (2015), Zhang et al. (2016)
		E	It modulates the neurotransmitter system and maintains therapeutic potential by preventing the activation of excess extrasynaptic N-methyl-d-aspartate (NMDA) receptors	Hussain et al. (2018), Ciechanover and Kwon (2015)
Berberine, epiberberine, coptisine, palmatine, groenlandicine, jateorrhizine, magnoflorine	*Coptis chinensis* Franch	AD	They can inhibit A*β* aggregation by preventing the metabolic pathway that constitutes A*β* plaques. These compounds also present AChE inhibiting effects and antidepressant activities and improve cognitive enhancements. In addition, groenlandicine and jateorrhizine have important peroxynitrite scavenging activities, while coptisine and groenlandicine display moderate total ROS inhibitory effects	Park et al. (1996), Hsieh et al. (2000), Kong et al. (2001), Kim et al. (2004), Jung et al. (2009)
Caffeine (Methylxanthine derivatives)	*Coffea arabica* L.	AD	It decreases A*β* accumulation in the cortex and hippocampus and prevents the presinilin-1 and *β*-secretase levels. It potentially decreases neurotoxicity as it shows potential activity to act as an antagonist of adenosine receptors. Its consumption at a daily dose of 300–400 mg in tea, coffee, and soft drinks decreases the risk of cognitive impairment and AD development owing to its psycho-stimulant properties	Hussain et al. (2018), Laurent et al. (2014), McIntire et al. (2014), Nehlig (2016)
		PD	It shows its capability as an adenosine A2 receptor antagonist, resulting in enhanced locomotor activity in PD. Its ability to affect dopaminergic neurotransmission and attenuate neuronal cell death has been indicated. Moreover, it mediates dopamine receptor-mediated behavioral responses such as movement and cognitive functions. It leads to the down-regulation of adenosine A2 receptors to counteract the suppressive effect of adenosine on brain dopaminergic transmission	Hussain et al. (2018), More et al. (2013)
Conophylline (Vinca Alk.)	*Tavernaemontana divaricate* (L.) R. Br. ex Roem. & Schult. *Ervatamia microphylla* (Pitard) Kerr.	PD	It stimulates autophagy in an mTOR-independent manner. In rat pheochromocytoma PC12 cells, it inhibits protein aggregation and maintains cells from cell death caused by treatment with a neurotoxin 1-methyl-4-phenylpyridinium (MPP +) by inducing autophagy	Umezawa et al. (2018)
		HD	It removes mutant huntingtin aggregates	
Galanthamine (Isoquinoline Alk.)	*Galanthus woronowii* Losinsk. *G. nivalis* L. *Narcissus tazetta* L. *Leucojum aestivum* L.	AD	Unlike the semisynthetic derivatives, this alkaloid is a well-established drug for the treatment of AD and can be effective not only by itself but can also interact with other molecules. It affects the central cholinergic pathways and improves cholinergic neurotransmission by preventing AChE. It binds to AChE in the brain, by reducing the catabolism of ACh, increasing the level of ACh in the synaptic cleft. It was proven to develop cognition in patients with mild AD without symptoms of hepatotoxicity at the dose of 24 mg/d in clinical studies. It modulates nicotinic acetylcholine receptors (nAChR) activity, enhancing nicotinic neurotransmission, cognition, and memory. It shows positive stimulation in hippocampal neurogenesis via *α*7 nicotinic ACh receptors and prevents A*β* accumulation and cytotoxicity	Hussain et al. (2018), Kita et al. (2014), Cortes et al. (2020)
Geissospermine (Indole Alk.)	*Geissospermum vellosii* Allemão	AD	It develops cholinergic transmission with its AChE inhibitory property	Hussain et al. (2018), Araújo et al. (2011)
Harmine (Indole β-carboline Alk.)	*Peganum harmala* L.	AD	Due to its efficacy in penetrating the blood–brain barrier and the parenchyma cells of the brain and limiting the activity of AChE, it can effectively increase spatial cognition and also alter compromised memory through cholinergic neurotransmission improvement at a dose of 20 mg/kg. In addition to facilitating brain development and proliferation by inhibiting DYRK1A, it significantly intensifies tau protein phosphorylation by inhibiting DYRK1A catalyzed phosphorylation of tau. While high doses of harmine increased the BDNF protein level, which was reduced in depressive states, imipramine, a common anti-depressant drug, did not	Hussain et al. (2018), Moloudizargari et al. (2013), Klein-Junior et al. (2014)
		PD	Studies of both endogenous and exogenous beta-carbolines displayed that they have dopamine active transporter-mediated dopaminergic toxicity. In addition, beta-carbolines (harmine and harmaline) have a considerable effect against PD through inhibition of MAO-B	Moloudizargari et al. (2013)
Huperzine A (Lycopodium Alk.)	*Lycopodium serratum* Thunb.	AD	Its neuroprotective property in AD majorly involves mitochondrial protection from the toxicity induced by A*β* aggregation. It acts as an NMDA receptor antagonist, reducing glutamate-excite toxicity and minimizing the level of synaptic loss with neuronal cell death. It has been shown to reduce the BDNF level in patients with AD and mild cognitive impairment. It also presents AChE-inhibitory activity. It is clinically approved in China	Hussain et al. (2018), Shao (2015)
Lobeline (Piperidine Alk.)	*Lobelia inflata* L.	PD	It maintains dopaminergic neurons against 1-methyl-4-phenyl-1,2,3,6-tetrahydropyridine (MPTP) reducing nigral dopamin. It motivates reverse transport of dopamine from synaptic vesicles and prevents the uptake of dopamine itself into the synaptic vesicles via the vesicular monoamine transporter 2	Hussain et al. (2018)
Lycorine (Isoquinoline Alk.)	*Crinum asiaticum* L. *Hippeastrum morelianum* Lem. *H. psittacinum* Herb. *Narcissus broussonetii* Lag.	AD	It has a potential neuroprotective activity through the stabilization of radical species	Cortes et al. (2020)
Montanine (Isoquinoline Alk.)	*Hippeastrum vittatum* (L'Hér.) Herb.	AD	In a dose-dependent manner, it inhibits AChE	Hussain et al. (2018)
		E	It maintains against convulsions by modulating neurotransmitter systems, including GABAA receptors	
Morphine (Isoquinoline Alk.)	*Papaver somniferum* L.	AD	It increases GABA levels in the brain synapse, reducing A*β*-induced neurotoxicity by activating MOR in the CNS. It can reverse iA*β*-induced electrophysiological changes by protecting against intracellular A*β* (iA*β*) toxicity in main neuronal cultures	Hussain et al. (2018), Cui et al. (2011), Ye et al. (2014)
Nantenine (Aporphine Alk.)	*Nandina domestica* Thunb.	E	It is efficient in epilepsy owing to its capability to supply neuronal hyperexcitability by reducing calcium influx into the cell	Hussain et al. (2018)
Nicotine (Pyridine Alk.)	*Nicotiana tabaccum* L.	AD	Its potential in AD by upregulating the *α*4 and *α*7 nAChRs level is explained successfully. It has been reported to bind to the *α*-helix structure, limiting A*β*-peptide formation and also enhancing memory and learning mediated through neuropeptide Y receptors. Nicotine has been presented in various clinical trials to improve attention and memory in the treatment of AD patients. First, i.v. nicotine application developed cognitive functions, while another study found that two weeks of nicotine skin patch treatment followed by transdermal nicotine in a four-week trial, offered a significant improvement in cognitive functions	Girdhar et al. (2015), Hussain et al. (2018)
		PD		
Piperine (Piperidine Alk.)	*Piper nigrum* L. *P. longum* L.	AD	It inhibits AChE and *β*-secretase enzymes. It has been shown to act at a dose of 20 mg/kg in combination with quercetin and increase the neuroprotective effect of quercetin. In the ethylcholine aziridinium-induced AD model, which mimics the cholinergic hypofunction display in Alzheimer’s disease, it lowered the cognitive deficits and hippocampal neurodegeneration linked with this AD model. These effects may possibly be due to their antioxidant and anti-inflammatory activities	Fischer and Hanin (1986), Gupta et al. (2000), Hake et al. (2009), Chonpathompikunlert et al. (2010)
		PD	It inhibits the MAO B enzyme and increases the dopamine level. It also has potent anti-depressant activity. It has the effect of improving coordination and balance in 6-OHDA-induced parkinsonian rats	Hussain et al. (2018), Al-Baghdadi et al. (2012)
		E	It modulates serotonin, GABA, norepinephrine, and postpones tonic-clonic seizures by raising cortical and hippocampal serotonin and GABA levels	Hussain et al. (2018)
Physostigmine (Pyrroloindole Alk.)	*Physostigma venosum* Balf.	AD	It evolves cognitive abilities in normal and AD patients by inhibiting the BChE enzyme, however, it is now clinically not in use. Rivastigmine, a synthetic analog of physostigmine, has been reported to be therapeutically successful and has dual-action AChE- and BChE-inhibitory properties in clinical studies	
		PD	Phenserine is a physostigmine derivative that has the ability to reduce *α*-synuclein expression in neural cell lines	
Rhynchophylline, Isorhynchophylline (Tetracyclic oxindole Alk.)	*Uncaria rhynchophylla* (Miq.) Jacks.	AD	It has been reported to be useful because of its neuroprotective activity against neurotoxicity induced by A*β* by suppressing cellular apoptosis created by the mitochondrial pathway and prevention of oxidative stress. Moreover, it downregulates the levels of the protein Bcl-2/Bax ratio by decreasing A*β*25-35-induced neuronal apoptosis in the hippocampus	Hussain et al. (2018), Xian et al. (2012)
		PD	It reduces *α*-synuclein and maintains neuronal cells through the autophagy-lysosome pathway	Hussain et al. (2018); Zhang et al. (2017)
Salsoline (Isoquinoline Alk.)	*Salsola oppositifolia* Desf.	AD	It has the ability to inhibit the AChE and BChE enzymes, especially it is selective against BChE	Hussain et al. (2018)
Vincamine (Monoterpenoid indole Alk.)	*Vinca minör* L	ND	This compound increased cerebral blood flow, oxygen, and glucose utilization in neural tissue and supported the elevation of serotonin, dopamine, and noradrenaline levels. Besides, treatment of mice with vincamine at a daily dose of 15 mg/kg for 14 days lowered nearly 50% of their brain iron levels, suggesting a beneficial effect in reducing the oxidative stress linked with iron accumulation in neurodegenerative disease	Miyamoto et al. (1989), Fayed (2010)
Vinpocetine (Vinca Alk.)	*Vinca minor* L.	Ischemia Hypoxia	It adequately performs its potential to stimulate a wide variety of cell factors such as cytokines and chemokines leading to more activation of NF-κB. It stops the inflammatory response by overcoming the action of NF-κB from the cytoplasm to the nucleus. Thus, it reduces the cerebral infarction level by impeding necrosis and eventually plays a role as an anti-inflammatory agent. Because of its possible to cross the BBB and prevent Ca^2+^/calmodulin-dependent phosphodiesterase-1 and voltage-dependent Na^+^ channels, it has been utilized in ischemic stroke patients	Hussain et al. (2018), Wang et al. (2014)

### Flavonoids

Many studies have shown that flavonoids act as antioxidants and protect against cardiovascular diseases, some types of cancer, and age-related degeneration of cell components (Kumar and Pandey, 2013). The negative biological effects of flavonoid, which is consumed with our daily diet, are negligible (Middleton Jr et al., 2000). Chalcones act as a precursor to a wide variety of flavonoid derivatives found in plants. Mainly flavonoids comprise a six-membered heterocyclic ring to deliver a flavonone by a Michael-type nucleophilic attack of a phenol group to an unsaturated ketone (Dewick, 2002). Flavonoids have been reported to act on varied physiological and biochemical functions in the body, including antidepressant-like activity (Küpeli Akkol et al., 2019; Gürağaç Dereli et al., 2020). Studies show that antioxidant flavonoids have clinical potency in the treatment of depressive disorders and report that these secondary metabolites may be prototype agents in the research of new antidepressant drugs (Hritcu et al., 2017; Dereli et al., 2019).

Data from stress-induced depression models revealed that depression occurrence was intensely associated with increased monoamine oxidase (MAO) and malondialdehyde (MDA) amounts and glutathione peroxidase activities (Mao et al., 2009; Pláteník et al., 2014; El-Tarras et al., 2016). While the monoamine hypothesis is now seen as very simple to explain the complexity of the pathophysiology of depression, observations have contributed to the monoamine hypothesis, which suggests that depression is related to decreased norepinephrine (NE) and/or 5-hydroxytryptamine (5-HT levels) in the brain (Doboszewska et al., 2017). In addition, BDNF levels, the most frequently validated biomarkers, are low in depressive disorders (Pláteník et al., 2014). In this section, the influences of flavonoids on the monoaminergic system (brain‐derived neurotrophic factor) BDNF levels, and nerve cells, as well as antioxidant-related antidepressant effects are summarized.

The antidepressant-like effect of kaempferitrin (3,7-dirhamnoside of kaempferol) was examined in tail suspension test (TST) and forced swiming test (FST) in mice. Kaempferitrin, administered at a dose of 5, 10, and 20 mg/kg, produces an antidepressant-like effect associated with the serotonergic system, especially 5-HT1A. This activity was not associated with alterations in locomotor activity. Also, it is stated that a 1 mg/kg dose of kaempferitrin has a synergistic effect with imipramine and fluoxetine, but not with desipramine (Cassani et al., 2014).

The effects of flavonol quercetin and flavone luteolin were evaluated on MAO-A activity and protein level using SH-SY5Y nerve cells. After 24 h of incubation, while luteolin attenuates MAO-A activity, it has been stated that quercetin needs ascorbic acid to take effect. The study predicted that luteolin and quercetin did not significantly affect the MAO-A protein level, but they could be direct MAO-A inhibitors in SH-SY5Y cells by targeting mitochondria (Bandaruk et al., 2014). The potency of quercetin was assessed on the BDNF gene expression in the rat brain. Rats were treated with quercetin 10, 20, and 50 mg/kgbwt doses during 30 days by gastric gavage. Quercetin at 20 and 50 mg/kg doses lead to a meaningful increase in mRNA expression of BDNF toward the control group but had no effect at the 10 mg/kg dose. It has been noted that the neuroprotective activity of quercetin may be due, at least in part, to the inductive effects of BDNF mRNA on expression levels (Rahvar et al., 2018). In a bioassay-guided fractionation assay rutin, quercetin, quercitrin, and isoquercitrin were isolated from *Melastoma candidum.* The IC50 estimate of rutin, quercetin, quercitrin, and isoquercitrin on MAO-B were reported as 3.89, 10.89, 19.06, and 11.64 µM, respectively (Lee et al., 2001). MAO-A inhibition of quercetin was reported by van Diermen et al. (2009) with an 18 µM IC50 value (van Diermen et al., 2009).

A flavone C-glycoside vitexin, investigated for its anti-depressant-like activity in BALB/c mice. In the plus-maze, hole board, or activity cage tests, vitexin did not show meaningful changes in the behavior of mice at a dose of 10–30 mg/kg, but in TST and modified forced swimming tests (MFST) vitexin was found to reduce the immobility time. The anti-depressant-like activity of vitexin has been demonstrated through interference with the noradrenergic α (Lyketsos et al., 2007), dopaminergic D(1), D(2), and D(3), and serotonergic 5-HT (1A) receptors, and enhancement catecholamine amounts in the synaptic cleft (Can et al., 2013). Neuroprotective effect of vitexin is shown via suppression of activation of isoflurane-induced caspase-3, and enhanced levels of *p*-secretase 1 in PC12 cells. It has also been noted that vitexin reduced the amounts of isoflurane related cytosolic calcium and ROS (Chen et al., 2016). Another flavon-C- glycoside orientin was researched in chronic unpredictable mild stress model in mice. In total, 20 and 40 mg/kg doses of orientin were administered to mice by oral gavage. Treatment with orientin weakened oxidative stress markers and enhanced norepinephrine and serotonin concentrations in the hippocampus and the prefrontal cortex of chronic unpredictable mild stress mice. Orientin administration also augmented the BDNF and synapse-related proteins (Liu et al., 2015).

Naringenin (4, 5, 7-trihydroxyflavanone) was evaluated in the mouse behavioral models of depression by Yi et al. The effect of pretreatment on the anti-immobility effect of naringenin with *p*-chlorophenylanlanine methyl ester or α-methyl-*p*-tyrosine, serotonin, or noradrenaline synthesis inhibitors was investigated in the study. Naringenin significantly reduced immobility time after acute treatment in the mouse TST at doses of 10, 20, and 50 mg/kg. According to the outcomes of the study, naringenin has been reported to have a strong antidepressant-like feature through central noradrenergic and serotonergic systems (Yi et al., 2010). In a different study carried out by Olsen et al. (2008), the IC50 values of naringenin on MAO-A and MAO-B were as follows 955 ± 129 M and 288 ± 18 M (Olsen et al., 2008).

Two behavioral models, *p*-chlorophenylalanine stimulated depletion of serotonin and reserpine-stimulated hypothermia and ptosis, have been appointed to investigate the antidepressant effect of fisetin (3,3′,4′,7-tetrahydroxyflavone). The results showed that doses of fisetin 10 and 20 mg/kg (p.o) inhibit the duration of immobility in behavioral tests, and the doses affecting the immobile response did not affect locomotor activity. In addition, it was stated that 14.7% inhibition on MAO activity was found in the mouse brain and MAO-B activity was not affected with fisetin treatment (Zhen et al., 2012).

Apigenin has been demonstrated to have antidepressant effects. Inhibition of MAO activity, reduced GABA and NMDA receptors function, decreased immobility time in the FST with an increase in dopamine turnover in the hypothalamus has been proven by different studies (Lorenzo et al., 1996; Skolnick, 1999; Nakazawa et al., 2003; Han et al., 2007). To better understand the behavioral effects of chronic oral apigenin treatment, FST was performed in exposed male ICR mice and male Wistar rats. Apigenin attenuated changes in serotonin (5-HT), its metabolite 5-hydroxyindoleacetic acid (5-HIAA), dopamine (DA) levels, and 5-HIAA/5-HT chronic ratio in different rat brain regions. Treatment of oral apigenin has a combination of multiple biochemical effects and has been reported to help to clarify the mechanisms of action that play a role in the normalization of stress-stimulated alterations in brain monoamine levels (Yi et al., 2008). On behavioral changes, insulin signaling and CREB-BDNF axis in the hippocampus of high-fat, high-fructose-diet-fed rats were used to evaluate apigenin’s antidepressant effect. The experimental results have revealed that apigenin significantly improves Glucagon-like peptide levels, cognitive function, insulin signal, and affects the CREB (cAMP response element-binding)-BDNF axis. The binding of the CREB protein to the promoter IV of the BDNF gene was increased in animals that received apigenin (Kalivarathan et al., 2020).

The behavioral, neurochemical, and neuroendocrine effects of icariin studied in detail by Pan et al. Mice were administered icariin at 8.75, 17.5, 35, and 70 mg/kg doses by gastric gavage. It was found that icariin decreases the immobility time both in the TST and FST for 21 days. Also decrease in MAO-A and B levels, serum corticotropin-releasing factor amounts, and an increase in brain monoamine neurotransmitter amounts after icariin treatment were reported (Pan et al., 2005). Liu et al. conducted a study for evaluating the icariin’s antidepressant effect in chronic mild stress rats. According to the sucrose preference test and the FST, icariin decrease oxidative-nitrosative stress markers, interleukin-1β (IL-1β), and tumor necrosis factor-alpha (TNF-α) levels and inhibited the activation of the nuclear factor kappa B (NF-κB) signaling pathway. The study specified that icariin exhibits antidepressant effects, at least in part that may result in enhanced antioxidant status and anti-inflammatory activity through inhibition of NF-κB signal activation and NLRP3-inflammasome/caspase-1/IL-1β axis (Liu et al., 2015). The metabolite of the icariin, icaritin, has been shown to produce an antidepressant effect in mice. Treatment with icaritin at 5 and 10 mg/kg doses reverses the progress of social aversion after defeat stress. GR mRNA and BDNFmRNA expressions increased in the hippocampus after icaritin application and partly repaired social defeat-stimulated deterioration of glucocorticoid sensitivity (Wu et al., 2013).

The probable antidepressant activity of hesperidin was analyzed in mice with FST and TST. Mice were administered with intraperitoneal hesperidin at 0.1, 0.3, and 1 mg/kg doses. The outcomes revealed that hesperidin alleviated the duration of inactivity in FST and TST without affecting the locomotor activity in the open field test. The antidepressant-like activity caused by hesperidin in mice in TST has been linked to its interference with serotonergic 5-HT1A receptors (Souza et al., 2013). Hesperidin treatment (50 mg/kg, bw) in irradiated rats significantly attenuated oxidative stress, monoamines changes, and mitochondrial detriment in the cerebral hemispheres (Said et al., 2012).

Filho et al. (2015) disclosed the antidepressant activity of chrysin in mice exposed to chronic unpredictable mild stress. The study suggests that the antidepressant effect of chrysin may be associated with upregulated BDNF levels in the hippocampus and prefrontal cortex of stressed mice (Filho et al., 2015). A different study carried out by Filho et al. recommended a connection present between the antidepressant-like action of chrysin and the pro-inflammatory cytokines synthesis, 5-HT metabolism, kynurenine pathway, and caspases activities (Filho et al., 2016).

In a different study, MAO inhibition effects of anthocyanidins were performed and, MAO-A and MAO-B inhibitor IC50 values are as follows: pelargonidine (28 μM; 45 µM), peonidine (41 μM; 25 µM), malvidin (32 μM; 20 µM), delfinidin (36 μM; 38 µM), cyanidine (31 μM; 33 µM), and petunidine (35 μM; 45 µM) (Dreiseitel et al., 2009). Methanol extract of *Hibiscus rosa-sinensis* Linn. containing anthocyanins was examined for antidepressant activity in mice by employing behavioral tests such as TST and FST. In this study, the extract showed a significant reduction in immobility time in TST and FST, analogous to imipramine. Anthocyanin-rich extract has shown potential antidepressant activity through dopaminergic, noradrenergic, and serotonergic mechanisms (Shewale et al., 2012).

With many studies, the use of these flavonoids, which have an antioxidative nature, has been proven to be beneficial, in particular, to reduce symptoms of depression by different mechanisms. These mechanisms are: 1) regulation of different neurotransmitters such as dopamine, serotonin, and norepinephrine, 2) antioxidant characteristics, 3) anti-inflammatory effect, 4) capacity to regulate the activity of MAO enzyme, and 5) effect on neurotransmitter receptor systems.

Flavonoids have been established to be very selective anxiolytic compounds that do not have additional CNS effects. In the literature, there are examples of flavonoids as ligands for benzodiazepine receptors: isoflavans equol and 3′,7-dihydroxyisoflavan, and the biflavonoid amentoflavone (Luk et al., 1983; Nielsen et al., 1988). These secondary metabolites are inactive *in vivo*, but chrysin showed an anxiolytic effect at 1 mg kg^−1^ i.p. dose in mice (Wolfman et al., 1996). The anxiolytic effects of different flavonoids are summarized in Table 2.

**TABLE 2:** The anxiolytic effects of different flavonoids.

Flavonoid	Animal	Dose	Effect	References
5-Methoxy-6, 8-dibromoflavanone 6-bromoflavanone	Mice	0.5, 1 mg/kg (i.p)	All synthetic flavonoids increased locomotor activity and animal exploration skills in open field and hole board tests	Ognibene et al. (2008)
6-C-glycoside-diosmetin Isovitexin Vicenin-2 Vitexin	Rat	0.1 and 0.25 mg/kg	Isovitexin and 6-C-glycoside-diosmetin administration displayed memory enhancing and anxiolytic-like behaviors	Oliveira et al. (2018)
6-Methoxyflavanone	Rodent	10, 30 and 50 mg/kg	6-Methoxyflavanone applied an anxiolytic-like effect, increasing inputs and time spent on the open arm and central platform	Akbar et al. (2017)
Anthocyanin	Rat	200 mg/kg	Treatment with anthocyanin had an effect on its own and prevented anxiogenic behavior caused by streptozotocin	Gutierres et al. (2014)
Apigenin 7-glucoside	Rat	10 mg/kg	In the apigenin 7-glucoside treated groups, decreased time spent in closed arms and head dips in the EPM test	Kumar and Bhat (2014)
Baicalein	Mice	0.02, 0.2 pmol	Baicalein demonstrated an anxiolytic-like effect at low doses, increased the time spent in open arms and head dipping, reduced tense participation postures in the raised plus maze	de Carvalho et al. (2011)
Chrysin	Rat	0.5, 1, 2, and 4 mg/kg	Reduced anxiety-like behavior in both EPM and light/dark test at 2 and 4 mg/kg doses	Rodríguez-Landa et al. (2019)
Genistein	Rat	0.045, 0.09, and 0.18 mg/kg (s.c)	At the doses of 0.09, 0.18 mg/kg decreased anxiety-like activity in the EPM and also enhanced the time spent grooming and rearing	Rodríguez-Landa et al. (2017)
Gossypin myricitrin naringin	Mice	1–30 mg/kg (i.p.)	Myricitrin (1 mg/kg) was effective in the EPM test, which showed a clear anxiolytic effect without any sedation symptoms. Gossypin and naringin similarly caused a strong anxiolytic effect at low doses (1 mg/kg)	Fernandez et al. (2009)
Kaempferol	Mice	0.02–1.0 mg/kg (p.o.)	The anxiolytic effect of kaempferol was partly antagonized by co-administration of flumazenil	Grundmann et al. (2009)
Luteolin	Rat		Luteolin did not form anxiolysis by modulation of the GABAA receptor, but it was able to regulate motor movements and mobility	Raines et al. (2009)
Quercetin	Mice	1.25, 2.5, 5, and 10 mg/kg	At a dose of 5 mg/kg, quercetin leads to a significantly longer time to be spent on the open arms of the EPM and significantly increased entry percentages compared to the control group	Jung and Lee (2014)
Rutin	Rat	30–1000 mg/kg (i.p)	Rutin showed anxiolytic-like activity similar to diazepam	Hernandez-Leon et al. (2017)

### Phenolic Acids

The shikimate pathway enables an alternative route to aromatic compounds, especially the aromatic amino acids: L-tryptophan, l-tyrosine, and l-phenylalanine. A central intermediate in the pathway is shikimic acid. Phenylalanine and tyrosine form the basis of C6-C3 phenylpropane units e.g. coumarins, cinnamic acids, flavonoids, and lignans. Also, it is known that many simple benzoic acid derivatives (C6-C1) such as gallic acid and *p*-aminobenzoic acid are generated through branching points in the shikimate pathway (Dewick, 2002).

The antidepressant potency of gallic acid treatment in two different studies has been linked to preventing a stress-induced increase in MAO-A activity, MDA amounts, and plasma corticosterone and nitrite levels (Yen et al., 2002; Chhillar and Dhingra, 2013). To analyze the antidepressant effect of gallic acid TST and the MFST were applied to the mice. Reduced duration of inactivity in both TST and MFST without causing any change in the locomotor activity was shown in gallic acid (60 mg/kg) administered mice. The authors noted that gallic acid has a dual effect on the synaptic clefts of the CNS, by enhancing serotonin and catecholamine levels. In addition, alpha-adrenergic, 5-HT2A/2C, and 5-HT3 serotonergic and D1, D2, and D3 dopaminergic receptors also played a role in this antidepressant-like activity (Can et al., 2017). Interestingly Pereira et al. notified the anxiolytic but not the antidepressant-like effect of gallic acid. Gallic acid treatment was capable to generate an anxiolytic-like activity in the EPM and light-dark transition (LDT) tests, but not antidepressant-like effect in the FST. Also, oxidative stress in the rat hippocampus and prefrontal cortex was reversed with gallic acid via decreasing lipid peroxidation and increasing reduced GSH (Pereira et al., 2018).

To understand the antidepressant-like effect of aspirin and 3-hydroxybenzoic acid (3, 10, 30, 100, and 300 mg/kg, *p.o*.); TST, for 20 mg/kg daily oral doses of aspirin and mono-hydroxybenzoic acids; EPM and FST were performed in foot shock stressed rats. The results were also compared with the diazepam control group (5 mg/kg/day, *p.o*). Aspirin and all mono-hydroxybenzoic acids exerted stress response desensitizers, but only low-dose aspirin and salicylic acid had diazepam-like activities in stressed rats. In contrast to mono-hydroxybenzoic acids, aspirin has been reported to exhibit activities similar to antidepressants in stressed mice and rats after low oral doses (Ruipérez et al., 2010; Khan et al., 2015).

FST was performed to analyze the antidepressant potency of vanillic acid and its relationship with the Akt, ERK, and mTOR signaling and upstream α-amino-3-hydroxy-5-methyl-4-isoxazolepropionaic acid receptor (AMPAR) in the prefrontal cortex of mice. Vanillic acid exhibited antidepressant activity by considerably lowering behavioral disorder in FST. Besides, it was stated that vanillic acid augmented AMPAR efficiency, Akt, and mTOR signal, but did not increase ERK signal in the prefrontal cortex. According to the experimental results, the authors have revealed an Akt-dependent but ERK-independent mechanism underlying the antidepressant activity of vanillic acid, which may be helpful in some depression patients (Chuang et al., 2020). After treatment with vanillic acid (10, 30, and 100 mg/kg) for 2 weeks, chronic cerebral hypoperfusion was induced in rats. Pretreatment with vanillic acid improved sensory-motor signs and anxiolytic behavior compared to the control group (Khoshnam et al., 2018).

The antidepressant and anxiolytic effects of cinnamic acids with samples such as caffeic acid, *p*-coumaric acid, sinapic acid, and ferulic acid with the C6-C3 skeleton are summarized in Table 3.

**TABLE 3:** The antidepressant and anxiolytic effects of cinnamic acids.

Compound	Animal	Dose	Effect	References
Caffeic acid	Mice	1, 2 and 4 mg/kg (i.p)	Caffeic acid administered at a dose of 4 mg/kg decreased the duration of immobility in the FST	Takeda et al. (2002)
		4 mg/kg (i.p)	Duration of immobility decreased by caffeic acid. It has been noted that indirect modulation of the alpha1A-adrenoceptor system may cause antidepressive and/or anxiolytic-like effects of caffeic acid	Takeda et al. (2003)
	Rat	0.5, 1, 2, 4 or 8 mg/kg (i.p)	Caffeic acid (1 mg/kg) increased the number of entries and the time spent in the open arms on EPM, suggesting an anxiolytic‐like effect when used in lower doses without affecting locomotor activity on the open field	Pereira et al. (2006)
Ferulic acid	Mice	0.001, 0.01, 0.1, 1 and 10 mg/kg (p.o)	Ferulic acid showed antidepressant-like effects in FST and TST (0.01–10 mg/kg). Sub-effective dose of ferulic acid (0.001 mg/kg) formed a synergistic antidepressant-like effect in the TST with fluoxetine, paroxetine, and sertraline	Zeni et al. (2012)
	Rat	25 and 50 mg/kg (p.o)	Ferulic acid reduced immobility time, increased locomotor activity in FST. Ferulic acid inhibited serotonin, norepinephrine, and dopamine reuptake, regulated the HPA axis, increased ghrelin, and simultaneously stimulated jejunal contraction	Zhang et al. (2011)
		12.5, 25, and 50 mg/kg (p.o.)	Increased sucrose intake, and decreased immobility time, and the total number of crossings, rearing, and grooming. Ferulic acid inhibited IL-6, IL-1β, and TNF-α, and increased IL-10	Zheng et al. (2019)
	Mice	0.01, 0.1, 1 and 10 mg/kg/(p.o)	In the groups treated with ferulic acid, increased CAT, SOD activities, and decreased TBARS levels in the hippocampus were reported	Lenzi et al. (2015)
*p*-Coumaric acid	Rat	50,75 and 100 mg/kg (p.o)	*p*-Coumaric acid (50 mg/kg) decreased immobility time in FST and TST, also avoided the rise of inflammatory cytokines, and reduced BDNF in hippocampus	Lee et al. (2018)
	Rodent	3,10,30 and 90 mg/kg (p.o.)	A significant anxiolytic effect is promoted in EPM by oral administration of *p*-coumaric acid	Scheepens et al. (2014)
Sinapic acid	Mice	1, 2, 4 and 8 mg/kg (p.o)	Synapic acid (4 mg/kg) considerably increased the time spent on the open arms of the EPM test, and significantly increased the number of head dips in the hole board test. Anxiolytic-like activities are mediated via GABA(A) receptors and activating Cl (-) currents	Yoon et al. (2007)

The conducted researches reveal that the antidepressant activities of these cinnamic acids come into existence via substantial neurotransmitters such as serotonin, and besides by the participation of inflammation-associated metabolites such as AA-COX-2/5-LOX and BDNF (Diniz et al., 2019).

### Terpenes

Terpenoids, especially essential oils are the main compounds of the plant kingdom and form the largest group of phytochemicals. They are attributed to the pharmacological properties of a variety of medicinal plants (de Las Heras and Hortelano, 2015). They have analgesic, anti-inflammatory, anti-insomnia, antidepressant, anxiolytic, skin penetration enhancement, antiviral, antifungal, antibacterial, antiparasitic, cancer chemoprevention, and antihyperglycemic activities (Paduch et al., 2007; Gürağaç et al., 2018; Gürağaç Dereli et al., 2020; Gürağaç Dereli et al., 2020).

They are lipophilic and have a wide variety of domains including neurotransmitter receptors, muscle, and neuronal ion channels, G-protein receptors, cell membranes, enzymes, and second messenger systems. They work both individually and synergistically for many therapeutic effects. Additionally, they can increase the blood-brain barrier permeability (Baron and Baron, 2018).

Active triterpenoid saponins; asiaticoside, oxyasiaticoside, brahmoside, brahminoside, madecassoside, centelloside, isothankunoside, thankunoside, and related sapogenins (asiatic acid, etc) as well as monoterpenes; *β*-pinene and *γ*-terpinene isolated from *Centella asiatica* (CA) L. Urban, used for centuries in the Ayurvedic medical system as a “*medhya rasayna*” as a brain tonic, have improved, stimulated and strengthen the cognitive system and display antianxiety activity (Sakina and Dandiya, 1990; Nalini et al., 1992). Madecassoside decreases the depletion of dopamine and its metabolites, and the malondialdehyde (MDA) level significantly enhances the striatal BDNF level and modulates the ratio of Bcl-2/Bax in MPTP-induced PD in Wistar rats for Parkinson’s disease research (Shahpiri et al., 2016).

*Bacopa monnieri* has been used in the Ayurvedic medical system for centuries and its potent compounds bacoside A, bacopaside I and II, and bacosaponin C (Hou et al., 2002; Deepak et al., 2005) have anti-inflammatory and antidepressant effects (Sairam et al., 2002; Channa et al., 2006). Treatment with *B. monnieri* inhibited neuronal death by the prevention of AChE activity in primary cortical culture pretreated with A*β*25–35 (Limpeanchob et al., 2008). In addition, animals and volunteers treated with this plant showed improved memory (Stough et al., 2001; Das et al., 2002; Roodenrys et al., 2002).

*α*-Bisabolol is isolated from several plants such as chamomile flowers and has a neuroprotective effect (Nurulain et al., 2015). Borneol is obtained from camphor, rosemary, and mint. It has anti-insomnia and analgesic properties. It has an excellent blocking potential on the nicotinic acetylcholine receptor than lidocaine, suggesting it may be a stronger anesthetic (Park et al., 2003). Borneol has a developer for brain-targeting delivery systems (Zhang et al., 2013).

Camphor obtains mainly from the camphor tree, is easily absorbed by the skin, and creates a cooling sensation like menthol. Camphor has cholinesterase (ChE) inhibitory activity, beneficial in dementia and cognitive impairments (Perry et al., 2000).

*Rosmarinus officinalis* inhibits NO production (Lo et al., 2002) and protects dopaminergic neurons in different degenerative disease models (Kim et al., 2006; Lee et al., 2008; Park et al., 2008), probably due to its high amount of terpenes such as carnosol and carnosic acid (phenolic diterpenes). Carnosic acid increased neural cell viability, GSH levels, activation of the nuclear factor-E2-related factor 2 (Nrf2) pathway and ARE-luciferase reporter activity, as well as decreased cleaved caspase-3, poly (ADP-ribose) polymerase (PARP) activation, levels of reactive oxygen species, nuclear damage, enhanced JNK phosphorylation, and p38 activation in SH-SY5Y neuroblastoma cells exposed to 6-OHDA in Parkinson’s disease research (Shahpiri et al., 2016).

*β*-Caryophyllene is a known sesquiterpene found in numerous higher plants, especially in basil, black pepper, cloves, cinnamon, hops, rosemary, and oregano. It has antinociceptive effects in inflammatory and neuropathic pain (Klauke et al., 2014). It is a selective cannabinoid receptor type 2 (CB2) agonist. CB2 receptors have been included in the regulation of depression and anxiety-like behaviors, and *β*-caryophyllene has displayed antidepressant and anxiolytic-like effects in most widely used predictive animal models (EPM, OF, MBT; NSF, TST, and FST). *β*-caryophyllene administration decreased the anxiety-like behavior in the EPM test. In the OF test, it was anxiolytic as it enhances the time spent in the center area with no effect on total locomotor activity. Its treatment decreased the number of buried marbles supposing an anxiolytic-like activity in the MBT (Bahi et al., 2014). The study has proposed that CB2 receptors play a main role in alcohol reward and the CB2 receptor system seems to be included in alcohol (Al Mansouri et al., 2014) and cocaine (Xi et al., 2011) dependence and sensitivity via modulation of dopamine reward pathways. *β*-caryophyllene has been presented to decrease voluntary alcohol intake and attenuate ethanol-induced place preference and sensitivity in mice (Al Mansouri et al., 2014) as well as reduce cocaine self-administration (Xi et al., 2011). Thus, it can symbolize a possible pharmacological target for alcohol and cocaine abuse treatment.

Celastrol isolated from *Tripterygium wilfordii* lessened dopaminergic neuronal loss and suppressed DOPAC and dopamine level depletion in MPTP-induced PD in mice for Parkinson’s disease research (Shahpiri et al., 2016).

1,8-Cineole (Eucalyptol) is isolated from eucalyptus, bay leaves, tea tree, sweet basil, rosemary, common sage, wormwood, and mugwort. It is one of the terpenes reinforced by several randomized placebo-controlled trials. It has been demonstrated to develop memory and cognitive learning in accordance with the Natural Health Research Institute. Because of its ChE blocking activity, it has possible usage in AD (Perry et al., 2000; Moss and Oliver, 2012) and protection against *β*-amyloid-induced inflammation (Khan et al., 2014).

Cryptotanshinone, an abietane-type norditerpenoid quinone, is a potent compound of *Salvia miltiorrhiza* and has crossed the blood-brain barrier and decreased scopolamine-induced cognitive impairments *in vivo* (Kim et al., 2007). This compound also ensured useful effects in patients with ischemia and cerebral infarct (Adams et al., 2006). In addition, cryptotanshinone decreased A*β* aggregation in brain tissue by promoting amyloid precursor protein metabolism through the α-secretase pathway and developed spatial learning and memory in APP/PS1 transgenic mice (Mei et al., 2009).

The unique components of *Ginkgo biloba* have been determined as ginkgolides and bilobalide. The ginkgolides are diterpenes termed terpene trilactones with a cage skeleton made up of six 5-membered rings. They are antagonists of the platelet-activating factor (PAF) receptor. PAF is involved in the CNS including the modulation of long-term potentiation, an increase of intracellular Ca^+2^, and immediate early gene expression. Although the mechanism is unclear, the PAF receptor has recently been supposed to be a target for slowing the progression of neurodegenerative diseases, thus, it is a significant target in eliciting the neuromodulatory effects of terpene trilactones. In addition, ginkgolides were thought to be a selective and potent antagonist of glycine receptors, as long as they could exert their effect in the CNS. Clinically, benzodiazepines are used as anticonvulsants and anxiolytics and bind to receptors that are mainly found in peripheral tissues and glial cells in the brain. These receptors are known as peripheral benzodiazepine receptors (PBRs). The function of PBRs is unclear, but they have been supposed to play a role in cell proliferation, steroidogenesis, and stress and anxiety disorders. The second theory is supported by the increase in the number of PBRs in certain brain regions in neurodegenerative disorders and after brain injury. Various studies have displayed that the main effect of ginkgolides is the prevention of PBR expression. Additionally, many kinds of research have revealed that bilobalide affects the main neurotransmitters in the brain, such as GABA and glutamate. It has been shown to exhibit anticonvulsant activity against convulsions induced by 4-*O*-methylpyroxidine, pentylenetetrazole, and isoniazid. In rat cortical brain slices under hypoxic/hypoglycemic disorders, bilobalide significantly decreased glutamate release, assuming that the neuroprotective effects of bilobalide may be mediated by decreased glutamate efflux and, thus excitotoxicity. Therefore, possible medicinal applications in patients have been demonstrated, including the use of bilobalide to protect neurons from ischemia, as an anticonvulsant, and for anxiety treatment. In conclusion, there is an indication that the effect of these compounds on *β*-amyloid aggregation and potential protection against AD may be mediated by bilobalide at least in part. Recent findings also show that ginkgolides stop *β*-amyloid formation (Strømgaard and Nakanishi, 2004; Nakashi, 2005). Ginkgolide B suppressed the activity of caspase-3 to prevent neurotoxicity. It possessed a significant potential in restoring calcium-binding protein, calbindin D28K mRNA in PC12 cells exposed to 6-OHDA in Parkinson’s disease research (Shahpiri et al., 2016).

A series of tetracyclic triterpenoid saponins, ginsenosides, purified from *Panax ginseng* C.A. Meyer, is acetylcholine agonists and has improved learning and memory, cognitive and physical indolences involving actions on serotoninergic transmission, and dopamine neuron activity (Nocerino et al., 2020). Ginsenosides have presented a neuroprotective effect by increasing cell viability, inhibiting *α*-syn fibrillation and the seeding process of *α*-syn oligomerization and aggregation, enhancing oligomeric intermediate species stability, digestion by PK in BE(2)-M17 human neuroblastoma cells in Parkinson’s disease research (Shahpiri et al., 2016).

Huperzine A, a sesquiterpene alkaloid isolated from *Huperzia serrata* (Thunb. Ex Murray) Trev, the Chinese medicinal plant, displays a broad range of neuroprotective actions. Huperzine A has improved learning and memory impairments and ameliorated spatial working memory (Sakina and Dandiya, 1990; Nalini et al., 1992).

Specifically, Δ3,2-hydroxybakuchiol, a monoterpene, isolated from *Psoralea corylifolia* seeds used for years in Chinese medicine for cerebral aging and dementia treatment (Pérez-Hernández et al., 2016), maintained SK-N-SH cells from MPP + intoxication and inhibited the decrement of dopaminergic neurons in MPTP intoxicated mice by prevention of the monoamine transporter (Zhao et al., 2008; Zhao et al., 2009).

Isoborneol is explored in mugwort and other plants. It has antioxidant effects that may aid in neurodegenerative diseases such as PD linked with oxidative stress (Tian et al., 2007).

Limonene is found in the peels of all citrus fruits and is the second most common terpenoid in nature (Noma and Asakawa, 2010). Researches have presented properties including antidepressant and immunostimulating effects (Komori et al., 1995). Besides, limonene has been displayed to cause sleep and muscle relaxation (do Vale et al., 2002) being a potent anxiolytic in mice (Carvalho-Freitas and Costa, 2002). It decreased anxiety in patients with chronic myeloid leukemia (Pimenta et al., 2016). Limonene has been presented to increase the metabolic conversion of dopamine in the hippocampus and serotonin in the prefrontal cortex and striatum, suggesting that anxiolytic and antidepressant-like effects can consist of suppressing dopamine activity associated with increased serotonergic neurons through 5-HT1A (Komiya et al., 2006). Limonene displayed 10–12% enzyme inhibition for AChE and BChE at the maximum concentration of 2.03 mmol/L (Szwajgier and Baranowska-Wójcik, 2019).

Linalool is found in birch trees, coriander, citrus, lavender, laurels, rosewood, and many other plants. It presents properties like anti-nociception through activation of cholinergic and opioidergic systems (Peana et al., 2003), anti-anxiety/stress (Cline et al., 2008; Cheng et al., 2014), sedation (Buchbauer et al., 1991), anti-depressant, modulation of motor movements and locomotion (Cline et al., 2008), and anticonvulsant properties through anti-glutamatergic and GABA neurotransmitter systems (Elisabetsky et al., 1995; de Sousa et al., 2010).

Menthol is obtained from peppermint, corn mint, or other mint oils. It is a potent analgesic used topically for inflammatory pains such as joint, sprains, and muscle aches (Burkhart and Burkhart, 2003). Central and peripheral analgesic mechanisms involve activation of sensory neurons at the TRPM8 receptor, inhibition of voltage-gated sodium and calcium channels, selective activation of kappa-opioid receptors, and activation of GABA A receptors (Galeotti et al., 2002; Peier et al., 2002; Pan et al., 2012). Isopulegol is the precursor to menthol, and its properties cover antidepressant, antianxiety (Silva et al., 2007), and anticonvulsant effects (Silva et al., 2009).

Myrcene is a widespread monoterpene in aromatic plants such as bay leaves, sweet basil, lemongrass, parsley, wild thyme, tropical fruits such as hops, and mango. It has strong anxiolytic effects (Lorenzetti et al., 1991). The analgesic effects of myrcene were antagonized by naloxone suggesting an opioid-mediated mechanism (Rao et al., 1990). In addition, it has muscle relaxant, hypnotic, sedative (do Vale et al., 2002), and sleep aid effects (Bisset and Wichtl, 2004).

Nerolidol (*trans*-nerolidol) is explored in many plants including lemongrass, lavender, jasmine, ginger, oranges, tea tree, and low levels in orange and other citrus peels. It is approved by the US FDA as a food-flavoring agent. It has anti-insomnia and sedative effects (Binet et al., 1972).

The neuroprotection mechanisms of oleuropein, purified from *Olea europaea* leaf, increased cell viability and decreased biochemical markers of cell death, intracellular ROS level, Bax/Bcl-2 protein ratio, cleaved caspase-3 activation, and DNA fragmentation in PC12 cells exposed to 6-OHDA in Parkinson’s disease research (Shahpiri et al., 2016).

Paeoniflorin isolated from *Paeoniae alba* has neuroprotective activity by reducing *α*-syn accumulation via the increase in expression of LC3-II. It modulated acid-sensing ion channel currents resulting in increased cell viability. In MPTP-induced PD in C57BL/6 mice, paeoniflorin reduced dopaminergic neuronal loss and glial and astrocytic activation, inhibited iNOS, IL-1*β*, and TNF-*α*, and increased activation of the A_1_ adenosine receptor (Shahpiri et al., 2016).

Phellandrene is found in cinnamon, lavender, eucalyptus, grand fir, dill garlic, parsley, ginger, and water fennel. It has analgesic and anti-depressive properties (Piccinelli et al., 2015).

Phytol (acyclic diterpene alcohol) is a degradation product of chlorophyll and tocopherol. It is found in wild lettuce and green tea. It has anti-insomnia and relaxing effects and has been suggested to increase GABA levels via its inhibitory effect on succinic semialdehyde dehydrogenase (SSADH), one of the GABA degradative enzymes (Bang et al., 2002).

*α*-Pinene is the most common monoterpene in nature and is found in the aroma of fresh pine needles, conifers, and sage, as well as obtained from a variety of medicinal plants such as parsley, rosemary, basil, and dill (Noma and Asakawa, 2010). It is an AChE inhibitor that can aid memory and help counteract the short-term memory loss linked with THC. In addition, *Salvia lavandulaefolia* essential oil was investigated for *in vitro* inhibitory activity of human erythrocyte AChE compared with physostigmine and tacrine. The most potent monoterpenoids with total inhibition at 4.7 mM were *α*-pinene and 1,8-cineole among camphor, *β*-pinene, and bornyl acetate, etc. These results supposed that the inhibitory activity of *Salvia* essential oil has arisen from the synergistic effect of the constituents (Perry et al., 2000; Miyazawa and Yamafuji, 2005; Kennedy et al., 2011).

*Salvia miltiorrhiza,* a traditional Chinese plant, is known as Danshen and has been used for neurodegenerative diseases for thousands of years. Tanshinone I inhibited NO, IL-6, IL-*β*, NF-KB, TNF-*α*, and iNOS mRNA, GCSF production in murine BV-2 microglial cells exposed to LPS of *in vitro* Parkinson’s disease model. Besides, in *in vivo* models (MPTP-induced PD in C57BL/6 mice) tanshinone I decreased dopamine and its metabolites depletion and suppressed TNF-*α* and IL-10 (Shahpiri et al., 2016). Moreover, tanshinone IIA can improve learning and memory ability to be a viable candidate in the treatment of AD with mechanisms involving the ERK and glycogen synthase kinase-3*β* signal pathway (Lin et al., 2019). In another study, it was shown memory and hippocampal LTP improving the effect of tanshinone IIA in Alzheimer’s disease models. Tanshinone IIA evolved synaptic activation induced by BDNF (Li et al., 2016).

Terpineol and terpinolene are explored in eucalyptus, lilacs, pine trees, and lime flowers, and have anxiolytic and sedative properties (Buchbauer et al., 1991; Buchbauer et al., 1993; Ito and Ito, 2013).

*Valeriana officinalis* L. rhizomes contain valepotriates and sesquiterpenes (valerenic acid and acetoxyvalerenic acid). These compounds presented sedative effects in mice. In addition, valerian extracts have been shown to have GABA(B) receptor binding properties and GABA uptake inhibition in rat synaptosomes (Ortiz et al., 1999).

Sixteen terpenes were screened in *Drosophila* for AD models to describe their neuroprotective effects. Six of the 16 terpenes, *α*-pinene (+), and *β*-pinene (-), limonene (+), limonene (-), *ρ*-cymene and linalool, were partially suppressed the *β*-amyloid 42 (A*β*42). Among them, limonene (+) reduced the survival rate of flies expressing A*β*42 in neurons through development. Limonene (+) treatment did not affect A*β*42 accumulation and aggregation but caused cell death, reactive oxygen species levels, kinase phosphorylation regulated by extracellular signaling, and inflammation in the brains or imaginary discs of flies expressing A*β*42. This neuroprotective effect of limonene (+) was not linked with autophagic activity. Limonene has shown to have a neuroprotective effect against the neurotoxicity of A*β*42, and so it is a potent therapeutic agent for Alzheimer’s disease (Shin et al., 2020).

The anti-acetylcholinesterase activities of methyl jasmonate at 22%, nopol at 8.7%, 1,4-cineole at 21.4%, pulegone at 34.6%, carnosic acid at 37%, and (+)-menthol at 17.6% were shown. Besides, the anti-BChE activities of methyl jasmonate at 30.9%, nopol at 13%, 1,4-cineole at 22.2%, allo-aromadendrene at 13.2–17.4%, nerolidol at 15.6%, carnosic acid at 29.3%, isoborneol at 12.9%, and *β*-ionone at 47% were also determined (Szwajgier and Baranowska-Wójcik, 2019).

Considering all the experiments performed, including the TLC bioassay, Marston’s, and Ellman methods, the most promising AChE inhibitors are carvone, pulegone, and *γ*-terpinene together with the adequate activity of citronellal, farnesen, ocimene and *α*-phellandrene. The most favorable terpene that exhibits the highest AChE inhibitory activity and confirmed by tests was turned out to be carvone. Except for the *in vitro* studies, the molecular docking simulation disclosed that carvone was docked in the most important part of the AChE active site responsible for the correct functioning of the enzyme (Wojtunik-Kulesza et al., 2017).

A variety of terpenoids including Δ3,2-hydroxybakuchiol, pedicularioside A, and tenuigenin have shown neuroprotective activity by suppressing apoptotic enzymes (cleaved PARP and caspase-3), preventing dopaminergic neuronal loss, dopamine/norepinephrine uptake in synaptosomes, elevating the number of TH-positive dopaminergic neurons in *in vitro* and *in vivo* PD studies (Shahpiri et al., 2016).

Anxiolytic effects of promising compounds from plant-derived essential oils in animal models is summarized in Table 4.

**TABLE 4:** Anxiolytic effects of promising compounds from plant-derived essential oils in animal models (de Sousa et al., 2015; Zhang and Yao, 2019).

Promising compounds	Plant-derived	Administration way
(−)-Myrtenol	*Myrtus communis*	i.p.
(+)-Borneol	*Lavandula angustifolia; Thymus vulgaris*	
(+)-Cedrol	*Juniperus virginiana*	
(+)-Limonen	*Alpinia zerumbet; Citrus aurantium;C. limon; C. sinensis var. dulcis; Lippia alba; Thymus vulgaris*	
(+)-Limonen epoxide	*Citrus limon*	i.p., p.o.
2-Phenethyl alcohol	*Rosa centifolia; Rosa alba*	i.p.
Benzyl alcohol	*Cananga odorata*	Inhalation
Benzyl benzoate	*Cananga odorata*	
Camphene	*Abies sachalinensis; Lavandula angustifolia; Piper guineense; Thymus vulgaris*	i.p.
Carvacrol	*Origanum vulgare*	p.o.
Carvacryl acetate	*Satureja boliviana*	i.p.
Citronellol	*Pelargonium roseum; Rosa alba; Rosa centifolia*	
Farnesol	*Rosa damascena*	
Isopulegol	*Eucalyptus citriodora*	
Linalool	*Cananga odorata; Coriandrum sativum; Lavandula angustifolia; Ocimum basilicum; Piper guineense; Pelargonium roseum Thymus vulgaris*	Inhalation, i.p.
Linalool oxide	*Lavandula angustifolia; Ocimum basilicum; Pelargonium roseum*	Inhalation
Linalyl acetate	*Lavandula angustifolia*	
R-(−)-carvone	*Lippia alba*	i.p.
Safranal	*Crocus sativus*	
Thymol	*Thymus vulgaris; Lippia sidoides*	p.o.
*Valerena-4,7 (11)-diene*	*Nardostachys chinensis; Valeriana officinalis*	i.p.
*α*-asarone	*Acorus tatarinowii; Acorus gramineus*	i.p., p.o.
*α*-pinene	*Abies sachalinensis; Coriandrum sativum; Ducrosia anethifolia; Alpinia zerumbet; Stachys tibetica; Ferulago angulata*	Inhalation
*β*-caryophyllene	*Anthriscus nemorosa; Lavandula angustifolia; Ocimum basilicum; Spiranthera odoratissima; Thymus vulgaris*	p.o.

The lavender essential oil has been most properly investigated. It can relieve anxiety symptoms in three major application ways. Silexan is a capsule containing the higher ester type of *Lavandula angustifolia* essential oil. Germany registered this medicinal preparation for the treatment of anxiety. Many clinical studies have presented that Silexan can significantly alleviate anxiety symptoms in patients. *Citrus* essential oils have also been wildly used in clinical trials. *Citrus aurantium* oil has been found to lighten anxiety after oral or inhalation application. *Citrus bergamia* essential oil inhalation can construct the BP, HR, and HRV parameters on healthy volunteers. Inhaling *Citrus sinensis* oil decreased anxiety level and rehabilitated mood in dental patients. Limonene content varied in two studies of *Citrus aurantium* oil that have an anxiolytic effect. Various investigations reported that rose oil has physiological and psychological relaxation and anti-anxiety effects. Inhaling rose oil can reduce anxiety in women with primigravida. *Cananga odorata* oil has also shown great anxiolytic activity with its content of linalool and benzyl benzoate. In addition, it was displayed that carvacryl acetate content may differ in essential oil from *Satureja brevicalyx* and *S. boliviana* in anxiety relief studies by inhalation (Zhang and Yao, 2019).

Anxiolytic effects of promising compounds from plant-derived essential oils in clinical trials are summarized in Table 5.

**TABLE 5:** Anxiolytic effects of promising compounds from plant-derived essential oils in clinical trials (Zhang and Yao, 2019).

Plant-derived	Promising compounds	Administration way
*Lavandula angustifolia*	(+)-Borneol, Camphene	Inhalation (diffuser), oral, skin application (massage)
*Citrus aurantium; C. limon; C. sinensis* var. *dulcis*	(+)-Limonen	Inhalation (diffuser), oral
*Citrus limon*	(+)-Limonen epoxide	Inhalation (diffuser)
*Cananga odorata*	Benzyl alcohol, benzyl benzoate	Inhalation (diffuser), skin application (massage)
*Satureja brevicalyx, S. boliviana*	Carvacryl acetate	Inhalation (diffuser)
*Pelargonium roseum*	Citronellol	Inhalation (diffuser)
*Rosa damascena*	Farnesol	Inhalation (diffuser)
*Lavandula angustifolia; Cananga odorata; Pelargonium roseum*	Linalool	Inhalation (diffuser), oral, skin application (massage)
*Lavandula angustifolia; Pelargonium roseum*	Linalool oxide	Inhalation (diffuser), oral, skin application (massage)
*Lavandula angustifolia*	Linalyl acetate, *β*-Caryophyllene	Inhalation (diffuser), oral, skin application (massage)

**FIGURE 3:**
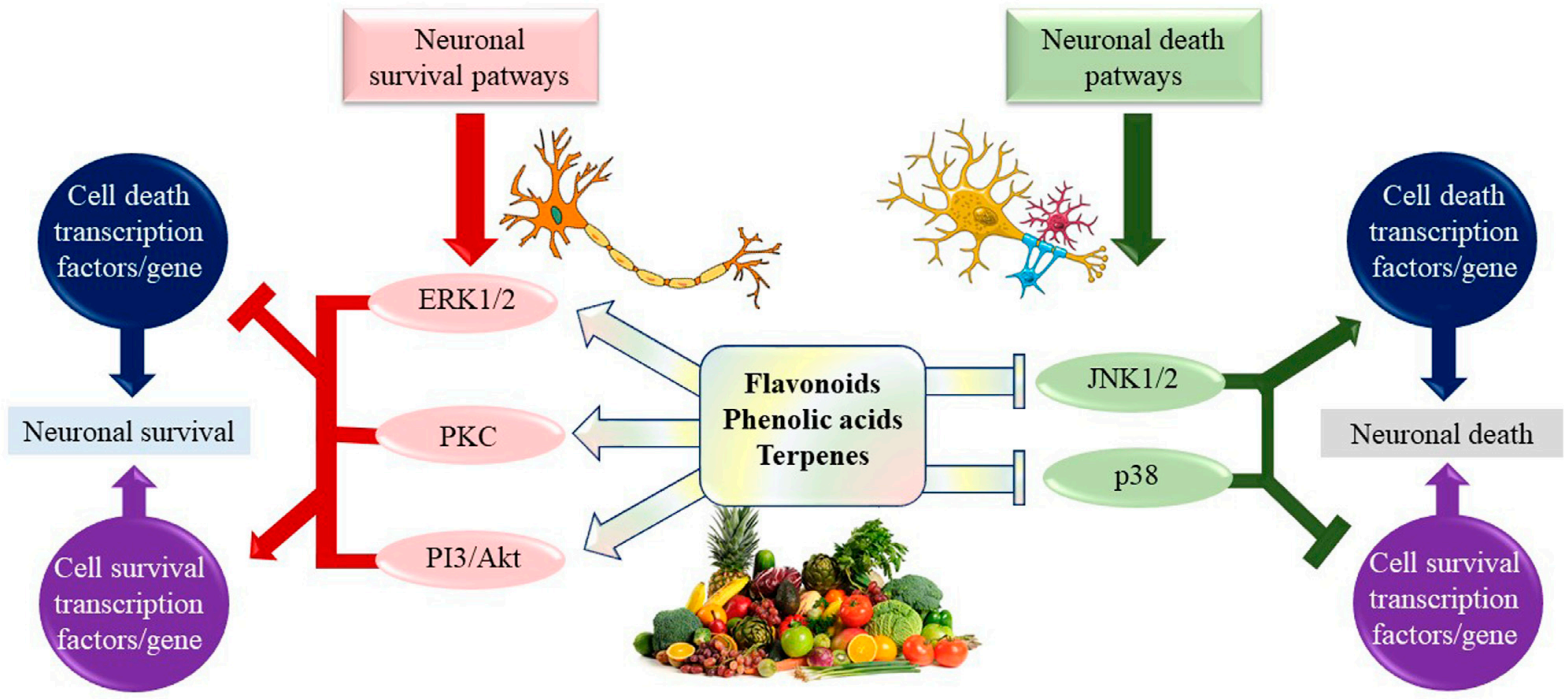
The modulation of neuronal survival and death protein kinase pathways via flavonoids, phenolic acids, and terpenes. The activation of ERK1/2, PI3K/Akt, and PKC pathways act to stimulate neuronal survival through the induction of pro-survival or antiapoptotic genes and by inhibition of pro-apoptotic proteins. JNK1/2 and p38 are stress-activated pathways and cause neuronal death. Furthermore, the inhibitory actions of flavonoids, phenolic acids, and terpenes in the JNK1/2 and p38 pathways are possible to be neuroprotective in the occurrence of stress signals. Though, flavonoids, phenolic acids, and terpenes have neuromodulatory and neuroprotective assets and prevent neuronal function by stimulatory and inhibitory actions at these signaling pathways.

The interaction of various flavonoids, phenolic acids, and terpenes in various ways and providing protection against neurodegeneration is summarized in Figure 3.

### Lipids

Lipids play a crucial role in neurons’ physiological functions and the structural development of the brain. The altered lipid composition of the brain is a significant risk factor for neurodegenerative disease. The vital role of lipids in tissue physiology and cell signaling in neurodegenerative diseases has been indicated, and some clinical studies have been conducted and completed for the efficacy of lipids on neurodegenerative disorders (Table 6) (Shamim et al., 2018).

**TABLE 6:** Clinical studies completed on lipids with protective effects of neurodegenerative impairments.

Lipids	Conditions	Dietary supplement
Alpha-linolenic acid	Effect of fatty acids on memory performance of toddlers	Flaxseed oil/Corn oil
Arachidonic acid (AA)	Autism spectrum, disorders	Aravita including AA + DHA
DHA	AD, autism, brain, cerebral, and mild concussion	DHA
	Learning, behavior	DHA/sunflower oil capsules
EPA	Subarachnoid, hemorrhage, cerebral vasospasm, schizophrenia, prodrome	Ethyl-EPA
	Major depression, dyskinesia, schizophrenia, schizoaffective disorder	EPA
PUFAs	ADHD	Eye Q
	Mild cognitive impairment, Alzheimer's disease	Omega 3 PUFAs (EPA + DHA)
Omega-3 fatty acid	Autism	Omega-3 fatty acid
	Depressive disorder, major	Omega-3 fatty acid/Corn oil
	Impaired psychomotor development	Preterm infants formula
	ADHD, reading/writing, disorder	Omega 3/6 fatty acids

The brain has the second-highest lipid concentration, after adipose tissue. The human brain is especially prospered in two PUFAs: docosahexaenoic acid (DHA, omega-3) and arachidonic acid (omega-6) that mammals should provide from the diet (Palacios-Pelaez et al., 2010). Monounsaturated fatty acids and polyunsaturated fatty acids (PUFAs) consumption has been demonstrated to decelerate cognitive reduction in animals and humans. Omega-3 and Omega-6 PUFAs are essential constituents of biomembranes and play significant roles in cell integrity, maintenance, development, and function (Shamim et al., 2018). DHA, a member of the essential omega-3 fatty acid family, is mostly found in fish oil obtained from salmon. Apart from marine sources, DHA can also be synthesized by desaturation from eicosapentaenoic acid (EPA), or in particular from *α*-linolenic acid, which is rich in *Salvia hispanica*, flax (*Linum* sp.), Soybean (*Glycine max*), and walnut. DHA is the most plentiful omega-3 PUFA in the brain and retina. DHA is enthusiastically captured and individually concentrated in the nervous system, especially in synaptic membranes and photoreceptors. DHA plays a significant role in aging, vision, neuroprotection, memory, and other functions. Up to 60% of all fatty acids esterified in neuronal plasma membrane phospholipid occur from DHA. Therefore, brain and retinal cells have also an accessible reservoir of esterified DHA. Esterified DHA has neuroprotective and anti-inflammatory properties. In addition, neuroprotectin D1 (NPD1) is the first defined oxygenated mediator from DHA to exert potent neuroprotective effects in the brain and retina. Supplementing DHA with carotenoid antioxidants such as lutein for neuronal disorders has been shown to considerably improve cognition and overall brain signaling functions (Crews et al., 2005).

A variety of researches have focused on omega-3 and omega-6 PUFAs found in nuts containing oleic acid, linoleic acid, and *α*-linolenic acid (Crews et al., 2005). Several studies have presented that consuming diets lacking in n23 fatty acids will disrupt cognitive function (McCann and Ames, 2005). Neuron’s structure is of great importance as the cells should keep proper electrical gradients through the membrane with normal anchor receptors and the ion channels to correspond with other cells and be able to deliver and reabsorb unmetabolized neurotransmitters. These properties rely on the fatty acid composition of the neuronal membrane. The fatty acid composition of neuronal membranes decreases through aging, however dietary supplementation with essential fatty acids has been presented to stimulate membrane fluidity and PUFA ingredients. As well as affecting membrane biophysical properties, PUFAs in the form of phospholipids in neuronal membranes can straight take part in signaling cascades to encourage synaptic plasticity, neuronal function, and neuroprotection (Yehuda et al., 2002; Kumar and Khanum, 2011).

Eukaryotic cell membranes consist of lipid bilayers with glycerolipids, their phosphorylated derivatives, and sphingolipids as main components. Phospholipids are essential for the biogenesis of autophagosomal structures. Therefore, the regulation of different phospholipid’s levels is important for this early autophagosome biogenesis. Dysfunction of autophagy causes the onset and progression of many neurodegenerative diseases. Progressive memory loss, amyloid *β* clusters in plaques, and hyperphosphorylated tau in the brain are distinguished properties of AD. Autopsy analysis of AD patients' brains has indicated a decrease in PIP2. This reduction is claimed to be caused by a result of increased levels of Synj1 well-known to dephosphorylate PIP2 during clathrin-mediated endocytosis. It is also presented that deregulation of PIP2 levels in *in vivo* AD models influences neurotransmission, spatial learning, and memory (Hernandez-Diaz and Soukup, 2020).

Unsaturated fatty acids, including linoleic acid, AA, *α*-linolenic acid, DHA, EPA, and oleic acid, are positively related with neuritic plaques and NFT charge and negatively associated with cognitive performance. In brain regions to AD pathology, there are decreases in linoleic acid, *α*-linolenic acid, and AA, and enhances in DHA. All these unsaturated fatty acids can interact directly with the A*β*40 and A*β*42 peptides and exhibit significant anti-aggregation properties by inhibiting amyloid fibril formation, particularly oleic acid and DHA. Therefore, the lipid-rich dietary supplement with the A*β*40 and A*β*42 antagonist-based method can be assumed to be effective for the palliative treatment of Alzheimer's (Shamim et al., 2018; Kao et al., 2020).

Recent studies have reported that the oligomerization of *α*-synuclein is stimulated by DHA. Researches have revealed that DHA levels are enhanced in PD brains related to controls, suggesting that DHA may play a role in the formation of insoluble *α*-synuclein clusters that do not show PD symptoms. Interestingly though, DHA shows that it can decrease the severity or postpone levodopa-induced dyskinesia development. Therefore, DHA may display a new approach to improving the quality of life of Parkinson's patients (Adibhatla and Hatcher, 2017; Shamim et al., 2018).

Oxidative stress plays an important role in the pathogenesis of HD, as in other neurological diseases, and oxidative stress is directly related to cholesterol metabolism. As HD progresses, it is clear that disturbances in cholesterol metabolism also progress, and that cholesterol metabolism products are a key indicator in HD. Together with, HD is caused by a repetitive trinucleotide in the Huntingtin (Htt) gene. Therefore, this situation has been attributed to the specific effects of the mutant Huntingtin gene on sterol regulatory element-binding proteins. Besides, protecting against motor deficiencies in a transgenic mouse model of HD by essential fatty acids supplementation has been presented. Moreover, EPA, DHA, and omega-3 fatty acids have been studied in clinical trials of HD patients. These studies displayed promise and the efficacy is hypothesized to be via the c-Jun amino-terminal kinase pathway. Researchers managed a 6-months randomized, placebo-controlled pilot study of the ethyl ester of ethyl-EPA (Miraxion™) in seven patients with Stage III HD. Patients treated with ethyl-EPA exhibited beneficial motor, MRI changes, and improvement in the orofacial component of United HD, after the 6-months study (Adibhatla and Hatcher, 2017; Shamim et al., 2018).

Recently, investigations on the neurological deficiencies of schizophrenia have been shown to focus on abnormalities in phospholipid metabolism, especially the stimulated activity of polylactic acid enzymes and the decreased activity of the system incorporating PUFAs into phospholipids. Monoamine oxidation plays an important role in the development of neurological disorders, DHA and EPA are substantial fatty acids for brain development, monoaminergic neurotransmission, and synaptic function. In addition, DHA and EPA enhance monoamine-mediated neurotransmission and inhibit disease progression. Therefore, supplementing essential fatty acids can ease symptoms of schizophrenia (Adibhatla and Hatcher, 2017; Shamim et al., 2018).

## Conclusion

Neurodegeneration is a process involved in both neuropharmacological disorders and brain aging. Cognitive dysfunction is a major health problem in the Twentieth century, and several neurodegenerative and neuropsychiatric disorders such as Alzheimer’s disease, depression, schizophrenia, cerebrovascular impairment, head injury, dementia, seizure disorders, and Parkinsonism can be severely functionally debilitating in nature.

Phytochemicals have been recently presented in several experimental models of neurological impairments for their neuroprotective effects. While the demand for phytotherapeutic agents is increasing, scientific verification is needed before plant-derived extracts and isolated compounds responsible for the activity, gain wider acceptance and use. Therefore, “phytochemicals” may ensure a new source of beneficial neurodegenerative drugs. Compared to conventional standards, most analyzed phytochemicals presented lower activity. This fact can be comprehended as a negative feature of these kinds of compounds but it should be noted that the main purpose of the studies is to find an active agent against multiple factors that may be responsible for the development of neurodegenerative disease. Moreover, a very important property for a suitable neuroprotective agent relates to its ability to cross the blood-brain barrier to reach the target sites of the CNS. Although the presence of receptors or transporters for phytochemicals in brain tissues is still being investigated, compounds with multiple targets seem to be an eventual and promising therapeutic class for the treatment of neuro impairments.
